# Analysis of biomechanical gait parameters in patients after total hip replacement operated via anterolateral approach depending on size of the femoral head implant: retrospective matched-cohort study

**DOI:** 10.1007/s00402-021-04264-6

**Published:** 2021-11-27

**Authors:** Artur Stolarczyk, Magda Stolarczyk, Łukasz Oleksy, Grzegorz J. Maciąg, Piotr Stępiński, Jakub Szymczak, Maciej Świercz, Krystian Żarnovsky, Marcin Mostowy, Bartosz M. Maciąg

**Affiliations:** 1 Department of Orthopedics and Rehabilitation, Medical University of Warsaw, Warsaw, Poland; 2 3rd Clinic of Internal Medicine and Cardiology, Medical University of Warsaw, Warsaw, Poland; 3 Medical University of Lodz, Lodz, Poland

**Keywords:** Total hip replacement, Arthroplasty, Gait, Large head, Biomechanics, Pattern

## Abstract

**Introduction:**

Total hip replacement (THR) is considered one of the most effective medical procedures in treatment of osteoarthritis. Since its introduction, there has been a worldwide debate over proper implant selection in terms of size, bearing type and shape. Following study was designed to assess the importance of femoral head size in long-term follow-up.

**Materials and methods:**

A cohort of 30 patients with primary end stage osteoarthritis who underwent total hip replacement was analysed retrospectively. A homogenous group was chosen with no major differences in BMI. Patients’ gait parameters were measured in a biomechanics laboratory using the 3D BTS Smart system. WOMAC and VAS questionnaires were used to assess patient reported outcome.

**Results:**

The subgroup with larger implant head size had several outcomes significantly superior to the subgroup with standard head size and non-inferior to healthy hips. Following variables were measured during this study: time of support phase, time of swing phase, double support time, walking hip extension angle.

**Conclusions:**

Use of larger sized femoral heads during THR gives better results in terms of gait pattern. Since restoring the gait pattern is one of the aspects of rehabilitation and returning to daily activities it seems to be an important observation.

## Introduction

Total hip replacement (THR) is considered to be one of the most effective medical procedures, being even named as the operation of the century [1]. It is estimated that the number of patients undergoing this surgery in the United States in 2020 will reach almost 500 thousand [2].

Since its introduction, there has been a worldwide debate over proper implant selection in terms of size, bearing type and shape [3–5]. Surgeons put effort into choosing the best combination of implant components to achieve personalization of the prosthesis and maximize the therapeutic effect of the surgical procedure. One of the most important aspects is femoral head size which has had a growing interest over the recent years. The average diameter of femoral head components used in THR grew throughout the years—from 22 mm in the 1960s to 32 mm in the 2000s, which is the most often used size nowadays. Over the recent years there was a notable number of large femoral heads (> = 36 mm) used in several registers [6–9]. There are many studies providing strong evidence that the range of motion, risk of dislocation, functional results, pain and prosthesis wear are dependent on femoral head size. Majority of them favour larger ones [10–19].

However, in the most recent reliable reviews, there are interesting observations made which make surgeons reconsider their decisions during THA. Studies prove that hip function and patient-reported outcome do not improve in THA with heads diameter between 32 to 36 mm. That should be considered along with evidence that hip range of motion increases with larger bearing sizes up to 36–38 mm, and also that 36 mm or larger femoral heads provide greater stability compared to 28 mm or smaller, and probably even to 32 mm [10, 17, 18].

There are also some complications after THA, which are being explained by larger femoral head use. One of them is a possible cause–effect relation between large femoral heads and taper corrosion but it is still controversial. Another controversy is the potential causative effect of larger head size on the incidence of groin pain due to iliopsoas impingement after THA. Since neither was clearly proven, both complications need further investigation [20–23].

There are several high-quality studies analysing factors mentioned above, but there is still a limited number of research concentrating on gait pattern after total hip replacement and its dependence on the implant head size. Since the femoral head size has a proven impact on range of motion, it is highly probable that it also alters the gait pattern or at least some parameters of gait that may be important in the rehabilitation process [18, 24].

Choosing adequate implants during total hip replacement might be crucial for improving the outcome and maximizing the results of the surgery. It might expedite restoring limb function and hip biomechanics, rehabilitation and help lower socioeconomic factors associated with total joint replacement.

Hypothesis of authors of this manuscript is that larger femoral heads allow to restore a healthy gait pattern due to the more anatomical femoral head size; thus, restoring more native hip biomechanics.

The aim of this study was to assess potential differences in lower limb biomechanics during gait in patients following primary total hip replacement surgery performed via anterolateral approach depending on femoral head diameter and to compare the results of the operated limb to the healthy one. As a secondary outcome authors wanted to inspect any correlation between gait parameters and patient-reported outcome and investigate possible superiority of larger femoral head implants over smaller ones in terms of postoperative gait biomechanics.

## Materials and methods

This study was conducted according to the Consolidated Standards of Reporting Trials (CONSORT) and an appropriate checklist was presented to the editors of the Journal [25]. This study was retrospectively registered on ClinicalTrials.gov (Registration number: NCT04521842). Institutional Ethics Committee approval was obtained and every participant signed a written consent to participate.

A consecutive series of 19 patients who received an uncemented Maxera Taperloc (Warsaw, IN, USA) metal-on-conventional-polyethylene THA system with head diameter of 36 mm between May of 2017 and June of 2017 was identified. Patients included in the study were: (1) aged > 60 years, (2) had BMI (kg/m2) < 40, (3) were able to walk for 10 m, (4) had leg length discrepancy < 5 mm, (5) knee flexion angle > 90 degrees, (6) hip extension angle < 0 degrees, and (7) hip flexion angle > 90 degrees, (8) complaining and radiologically confirmed single limb hip osteoarthritis, confirmed grade III and IV in Kellgren-Lawrence scale [26]. All participants received on-label use of an uncemented hip system as a treatment for end-stage hip osteoarthritis. Exclusion criteria included (1) patients with severe deformity with and (2) patients who underwent any other lower limb surgery before or after the THA, (3) patients with neurological disorders, (4) or severely impaired balance.

For the present analysis, the following demographic patient data were queried: sex, age at surgery (years), and BMI. A total of 16 patients treated with Maxera Taperloc (Warsaw, IN, USA) hip system met the inclusion criteria. All patients at the institution have a standard antero-posterior pelvic weight-bearing radiographic examination for evaluating intraarticular grade of osteoarthritis, leg discrepancy and assessment of hip joint alignment. Every patient fulfils the WOMAC questionnaire on the day of the admission to the hospital to assess hip joint function.

All surgeries were performed in a level III academic hospital. All operations were performed by an experienced total joint replacement surgeon.

All patients were operated in the lateral decubitus position. Surgical technique using natural interval in gluteal muscles and dissecting only one-third of its attachment was used. Incision in line with the axis of femoral shaft was performed with 1/3 distally and 2/3 proximally to the tip of the greater trochanter. Further blunt dissection of connective and fat tissue was done to visualize iliotibial tract. The latter structure was then incised in a slightly curved way so to stay in line with fibers of tensor fascia lata. After moving fascia aside, visualization of gluteus medius was done. Natural interval of 1/3 anterior part of the gluteus medius was found and carefully dissected from the bone. Then the femoral neck was easily palpable and the joint capsule was opened with a longitudinal dissection above the femoral neck. After completing the approach the hip joint was dislocated, the femoral neck was cut accordingly to manufacturer technique. All acetabular cups were templated in the position of 40–45 degrees inclination and 10–15 degrees anteversion to the supine anterior pelvic plane. All stems were uncemented. Proper prosthesis placement was confirmed on X-ray images taken on the following day. No leg discrepancy was observed.

Flexion and extension exercises of the ankle and isometric quadriceps contraction exercises were introduced on the first post-operative day, with full weight-bearing within pain tolerance. The duration of the exercises was 40 min to 1 h 3 times per day. All exercises were done bedside without using additional tools. The aim of mobilisation with a physiotherapist was to obtain flexion of the hip of 90°. Other methods of mobilisation included using a walker shoe and walking with crutches were introduced by the third day post-op. Patient-reported outcome (PRO) and gait pattern analysis of the large head diameter study cohort were compared to a 1:1 matched-control cohort of patients treated with the standard head diameter.

From May until June 2017, 16 patients underwent THA using the standard head diameter of 28–32 mm with use of Allofit Taperloc (Warsaw, IN, USA) hip system at our institution. For these patients, as well as the large-head diameter cohort, a propensity score based on age, sex, BMI, WOMAC, VAS score was generated.

Additionally, healthy volunteers (15 subjects) were recruited from the department employees’ families. All of them were examined prior to the gait analysis to exclude any lower limb pathologies and balance disorders. Healthy subjects from the control group and Allofit patients were matched to Maxera patients using a 0.1 propensity score threshold with priority given to exact matches.

All measurements were performed at least 3.5 years after the surgery (mean follow-up: 44 months). All patients underwent the same rehabilitation protocol in the same rehabilitation department immediately after the discharge from the orthopaedic ward. It was continued until the patient had the feeling that the function of their hip was restored to satisfactory level. Before gait pattern analysis all potential factors, such as comorbidities or pain, which might have influenced the results were excluded. Leg length and range of motion were measured in every case since these parameters might negatively affect the gait pattern, as proved in several studies [27–29].

## Gait analysis

Gait analysis was performed in University Biomechanical Lab using the BTS SMART Analyzer (BTS Bioengineering, Quincy, MA, USA) system for three-dimensional gait analysis by an experienced physiotherapist, with specialty in lower limbs biomechanics, who was unaware of patients implant size. All measurements and analysis were performed according to the Davis protocol [30].

Participants were asked to walk a 10-m distance in their normal tempo four times. During walking, their movement was recorded with use of markers placed on the base of the sacral bone, both anterior superior iliac spines, both greater trochanters, both lateral sides of the femur (half distance between greater trochanter and lateral femoral condyle), both sides on the fibular head, both lateral sides of the shin (half distance between head of the fibula and lateral malleolus), both bases of 5th metatarsal bone and calcaneal tuberosity.

Immediately before measurements, every participant was asked to walk through a marked route as many times as they wanted to feel fully comfortable with markers to minimize potential influence on their hip biomechanics. Measurements were performed and compared for both healthy and operated limbs of every patient (control group). Analysed parameters were time of support phase, double-support time, drop of contralateral side of pelvis during support, time of swing phase, length of step, mean walking speed, walking cadence.

## Patient-reported outcome

All participants fulfilled WOMAC (The Western Ontario and McMaster Universities Osteoarthritis Index) and VAS (Visual Analog Scale) questionnaires preoperatively during admission to the hospital and postoperatively during gait pattern analysis visit. The WOMAC questionnaire contains 24 questions concerning: pain, joint stiffness and physical functioning. The maximum result is 96, which represents the worst outcome [31–33]. The VAS score is a continuous scale consisting of a line for each symptom. A score of 0 represents “no pain” and a score of 10 represents “worst imaginable pain” [34].

## Radiographic evaluation

All patients preoperatively and at the final follow-up underwent radiographic examination in a supine position and with a 15° bilateral internal rotation of the hip joint with the center of the X-ray beam over the symphysis.

Cup inclination angles were measured on postoperative anteroposterior radiographs, as described in the study by Wan et al. [35]. Radiographic cup anteversion was measured with the method described by Lewinnek et al. [36] (femoral offset was measured as the perpendicular distance from the center of rotation of the femoral head to the central axis of the femur) [37]. Cup offset was measured as the horizontal distance from the center of rotation to the vertical tangent of Koehler’s teardrop’s lateral side [38]. Leg length was measured as the length of a vertical line drawn from the most prominent point of the lesser trochanter perpendicular to a horizontal line drawn between the two acetabular teardrops [39]. All measures were compared to the preoperative values and differences between groups were analysed.

## Statistical analysis

Results were analysed statistically. As all variables were continuous and the comparisons were performed between variables in unpaired groups, either Student’s *t* test for unpaired groups or Mann–Whitney *U* test were utilized, according to the normal distribution. Normality of distribution was tested using the Shapiro–Wilk test and the *p* value below 0.05 was considered significant.

## Results

A total of 15 patients from either large head diameter cohort (94%) or standard head diameter (94%) matched-control cohort completed the whole assessment at the final follow-up. In the large head diameter group one patient suffered from periprosthetic femur fracture due to the vehicle accident 2 years postoperatively, and in standard head diameter group one patient underwent two-stage revision hip replacement due to the late infection.

Mean age in large head diameter group was 70 years (SD = 9.52), while in standard head diameter group 68 years (SD = 10.87). Mean BMI (body mass index—kg/m2) in both groups was respectively 29.55 (SD = 4.52) and 29.53 (SD = 3.33) (Tables 1, 2, 3).

**Table 1:** Characteristic of participants in the large and standard head diameter matched cohort groups

	Participants characteristics			
	Large head	Standard head	Healthy volunteers	*p* value
BMI (body mass index—kg/m2)	29.55 (SD = 4.52)	29.53 (SD = 3.33)	29.49 (SD = 4.00)	> 0.05
Age (years)	70.0 (SD = 9.52)	68.0 (SD = 10.87)	69.0 (SD = 10.22)	> 0.05
Male:female	6:9	7:8	7:8	> 0.05
Right:left	10:5	11:4	10:5	> 0.05

**Table 2:** WOMAC scores preoperatively

WOMAC	Large head	Standard head	*p* value
Mean total	66.97 (SD = 10.23)	67.33 (SD = 12.11)	> 0.5
Mean function	43.66 (SD = 13.98)	44.60 (SD = 14.54)	> 0.5
Mean pain	11.47 (SD = 3.44)	11.73 (SD = 3.9)	> 0.5
Mean stiffness	4.33 (SD = 1.68)	4.66 (SD = 1.62)	> 0.5

**Table 3:** VAS preoperatively

VAS	Large head	Standard head	*p* value
Mean total	7.6 (SD = 2.12)	7.7 (SD = 2.23)	> 0.5

## Gait analysis

In group with standard head size, there was significantly higher time of support phase both in operated limb (72.3% vs. 61.0%, *p* = 0.012) and healthy limb (70.8% vs. 61.0%, *p* = 0.023); double-support time (20.3% vs. 13.0%, *p* = 0.001) as well as drop of contralateral side of pelvis during support both in operated limb (9.0 degrees vs. 7.0 degrees, *p* = 0.034) and healthy limb (8.5 degrees vs. 7.0 degrees, *p* = 0.036) compared with volunteers’ healthy hips. There was significantly shorter time of swing phase both in operated limb (27.7% vs. 39.0%, *p* = 0.007) and healthy limb (29.2% vs. 39.0%, *p* = 0.005); length of step both in operated limb (0.31 m vs. 0.73 m, *p* = 0.001) and healthy limb (0.44 m vs. 0.73 m, *p* = 0.021); lower mean walking speed (0.52 m/s vs.1.39 m/s, *p* = 0.007) and walking cadence (75.4 steps/min vs. 113.8 steps/min, *p* = 0.004) than in volunteers’ healthy hips (Table 4).

**Table 4:** Comparison of gait parameters between 28–32 mm femoral head and healthy hips (HH)

	Standard femoral head size		HH	*p* value	
	OL	HL		OL vs HH	HL vs HH
Support phase [%]	72.3	70.8	61.0	0.012	0.023
Swing phase [%]	27.7	29.2	39.0	0.007	0.005
Contralateral pelvic drop [°]	9.0	8.5	7.0	0.034	0.036
Stride length [m]	0.31	0.44	0.73	0.001	0.021
Double support [%]	20.3		13.0	0.001	
Mean gait velocity [m/s]	0.52		1.39	0.007	
Walking cadence [steps/min]	75.4		113.8	0.004	

*OL* operated limb, *HL* healthy limb

In group with large head size many more outcomes were restored to values not differing significantly from norms for healthy hips: time of support phase size both in operated limb (64.1% vs. 61.0%, *p* = 0.065) and healthy limb (64.0% vs. 61.0%, *p* = 0.064); time of swing phase both in operated limb (35.9% vs. 39.0%, *p* = 0.059) and healthy limb (36.0% vs. 39.0%, *p* = 0.06); double support time (16.4% vs. 13.0%, *p* = 0.057). However, drop of contralateral side of pelvis during support was higher in group with large head size than in healthy hips, both in operated limb (8.5 degrees vs. 7.0 degrees, *p* = 0.023) and healthy limb (8.0 degrees vs. 7.0 degrees, *p* = 0.046). Shorter than norms were: length of step of both operated limb (0.5 m vs. 0.73 m, *p* = 0.022) and healthy limb (0.6 m vs. 0.73 m, *p* = 0.041); mean walking speed (0.7 m/s vs.1.39 m/s, *p* = 0.025) and walking cadence (87.3 steps/min vs. 113.8 steps/min, *p* = 0.032) (Table 5).

**Table 5:** Comparison of gait parameters between 36 mm femoral head and healthy hips (HH)

	Large femoral head size		HH	*p* value	
	OL	HL		OL vs HH	HL vs HH
Support phase [%]	64.1	64.0	61.0	0.065	0.064
Swing phase [%]	35.9	36.0	39.0	0.059	0.06
Contralateral pelvic drop [°]	8.5	8.0	7.0	0.023	0.046
Stride length [m]	0.5	0.6	0.73	0.022	0.041
Double support [%]	16.4		13.0	0.057	
Mean gait velocity [m/s]	0.7		1.39	0.022	
Walking cadence [steps/min]	87.3		113.8	0.032	

*OL* operated limb, *HL* healthy limb

Both objective and subjective outcomes differed between the group with large head size and the group with standard head size. As to objective outcomes, the group with large head size had significantly shorter time of support phase than the group with standard head size both in operated limb (64.1% vs. 72.3%, *p* = 0.02) and healthy limb (64.0% vs. 70.8%, *p* = 0.015) and shorter double support time (16.4% vs. 20.3%, *p* = 0.027). The group with large head size had significantly greater time of swing phase both in operated limb (35.9% vs. 27.7%, *p* = 0.018) and healthy limb (36.0% vs. 29.2%, *p* = 0.03); stride length in operated limb (0.5 m vs. 0.31 m, *p* = 0.043); mean gait velocity (0.7 m/s vs. 0.52 m/s, *p* = 0.03); walking cadence (87.3 steps/min vs 75.4 steps/min, *p* = 0.011). There was no statistically significant difference in other analysed parameters (Table 6).

**Table 6:** Comparison of gait parameters between group with 28–32 mm femoral head and group with 36 mm femoral head

	Large femoral head size		Standard femoral head size		*p* value	
	OL	HL	OL	HL	OL	HL
Support phase [%]	64.1	64.0	72.3	70.8	0.02	0.015
Swing phase [%]	35.9	36.0	27.7	29.2	0.018	0.03
Contralateral pelvic drop [°]	8.5	8.0	9.0	8.5	0.09	0.086
Stride length [m]	0.5	0.6	0.31	0.44	0.043	0.12
Double support [%]	16.4		20.3		0.027	
Mean gait velocity [m/s]	0.7		0.52		0.03	
Walking cadence [steps/min]	87.3		75.4		0.011	

*OL* operated limb, *HL* healthy limb

## Patient-reported outcome

As to the patient-reported outcome measures, the group with large head size had significantly lower VAS score at rest (1.4 ± 0.7 vs. 2.23 ± 1.92, *p* = 0.041), Physical Function part of WOMAC score (15.18 vs. 24.96, *p* < 0.05) and WOMAC score as a whole (18.2 vs. 30.45, *p* < 0.05) (Tables 7, 8).

**Table 7:** WOMAC postoperatively

WOMAC	Large head	Standard head	*p* value
Mean total	18.20 (SD = 6.23)	30.45 (SD = 9.30)	< 0.05
Mean function	15.18 (SD = 6.83)	24.96 (SD = 13.36)	< 0.05
Mean pain	4.5 (SD = 2.59)	8.73 (SD = 4.05)	> 0.5
Mean stiffness	2.53 (SD = 1.67)	3.66 (SD = 2.01)	> 0.5

**Table 8:** VAS postoperatively

VAS	Large head	Standard head	*p* value
Mean total	1.4 (SD = 0.7)	2.23 (SD = 1.92)	< 0.05

## Radiological analysis

None of the analysed radiographic parameters differed significantly between the groups (Table 9). In both groups, one patient was identified as having the cup placed outside of the target zone.

**Table 9:** Radiological parameters postoperatively

Parameter	Large head	Standard head	*p* value
Cup anteversion [°]	12.20 (SD = 2.32)	12.45 (SD = 2.12)	> 0.5
Cup inclination [°]	41.78 (SD = 2.83)	42.46 (SD = 2.23)	> 0.5
Femoral offset [mm]	46.63 (SD = 4.44)	43.63 (SD = 3.23)	> 0.5
Cup offset [mm]	30.45 (SD = 4.00)	29.03 (SD = 2.03)	> 0.5
No. of patients with leg discrepancy > 5 mm	0	0	> 0.5

## Discussion

There are a few aspects on which we can assess the outcome of THR, most important among them being gait biomechanics restoration, patient-reported outcome, implant positioning and its wear.

In terms of gait characteristics, there are several deviations reported concerning both patients with hip osteoarthritis and following THR. It is well proven that those with hip OA have reduced stride length and reduced cadence, reduced gait velocity, and reduced joint excursion [24, 40, 41]. Patients after THR walk with lesser hip-abduction and sagittal-plane range of motion. It is believed that it might be a consequence of a pain-avoidance mechanism developed as an adaptation to joint disease. What is more, there are publications underlining that lower limb biomechanics during gait does not return to normal after THR [26, 42]. In one of these studies the follow-up was only about 11 months on average and participants were operated with lateral approach with one-third anterior and two thirds posterior hip adductors dissection. Authors in surgical technique dissect only one third using natural interval in gluteal muscles attachment. This technique allows for restoration of gluteal muscles in more than 50% of their native attachment. It seems that this enables faster rehabilitation and facilitates regaining full strength and length of the gluteal muscle tendon.

There is an ongoing debate about advantages and disadvantages of both standard and large femoral heads use during THA, concerning patient-reported outcomes, rates of dislocations, range of motion, bearing wear, taper corrosion, etc [3]. There is a limited number of studies analysing gait patterns after THA with use of different head sizes. The results of this study do not fully support conclusions made by Beaulieu et al. [26]. Several gait parameters of participants from large femoral head groups did not differ significantly from the healthy control groups. These are time of support phase both in operated and healthy limbs, time of swing phase and bipedal support time.

In this study, we noticed a significant correlation between gait pattern and the femoral head size used. Comparison between groups with different femoral head sizes performed in this study seems to carry no risk of bias, as the difference in VAS score during the double-support phase between them was statistically insignificant. These gait parameters may be important in case of rehabilitation. Since the aim of the treatment is to regain function and relieve pain, bringing back physiological gait pattern or at least making it more similar to physiological is an important step forward in achieving better functional results.

Physiological patterns have always been a reference for physiotherapists and our study seems to indicate a way to obtain better gait patterns. To our best knowledge, so far no papers favouring larger femoral heads with regard to gait pattern restoration in anterolateral approach THA have been published.

The study by Grip et al. [43], compared several movement patterns during walking, squats and stair climbing in groups with use of conventional head size implants (mean: 32.7 mm), large ones (mean: 53.5 mm) and control healthy group with use of wearable IMU-based motion analysis system. No significant differences were found when comparing gait parameters between large and conventional head size groups. The large femoral head group had significantly smaller average hip flexion–extension range of motion (ROM) during gait compared to controls. Significant differences in terms of range of motion parameters were also found between operated and non-operated limbs.. However, in this particular study participants were not randomized, no blinding was performed, thus potential risk of bias in this study might be higher. All patients included in this study were operated from a posterior approach. It is a strong limitation of the study since the size of the femoral head may reveal its effect on gait parameters in different approaches only.

In the study by Zagra et al. authors performed randomized-controlled trials comparing gait recovery between participants who received 28 mm, 36 mm, and ≥ 42 mm femoral head during THR [44]. Spatiotemporal gait parameters, kinematic or kinetic gait parameters were analysed during 4-months rehabilitation period. We believe that such period after the surgery is too short to fully evaluate gait pattern parameters after THA, as it was proven that functional outcome improves even up to 7 years following the surgery [45, 46]. No significant differences were observed between groups during this follow-up.

Additionally, in two studies mentioned above [26, 43], patients were operated via posterior approach. This approach is associated with irreversible damage to the posterior hip capsule, hip external rotators and pelvic stabilizers, what might be the reason for such results.

What is more, participants with large femoral heads had significantly better results in VAS and WOMAC score. Such results correspond partially to only one study by Matsushita et al. where researchers proved better functional results in daily living activities for total hip replacement with 36 mm head diameter [47]. However, in the most recently published systematic review [3] comparing 32 and 36 mm heads, it was suggested that there are no functional benefits of using larger heads. On the other hand, in two studies analysed in this publication [17, 18] participants were operated from the posterior approach, only one study was a prospectively randomized one, while in the other participants were operated by 17 different surgeons and were not randomized to receive a particular head size.

During the course of this study no prosthesis dislocation nor revision surgery due to other reasons were observed. In the systematic review [3], there was a lower risk of revision due to dislocation in the group with 36 mm head in comparison to 32- and 28-mm ones. This difference between the two studies might be due to the much smaller number of participants and shorter follow-up.

## Conclusions

This study is the first matched-cohort study to assess gait pattern parameters pre- and postoperatively in at least 3.5 years follow-up in patients undergoing total hip replacement with use of 28–32 mm and 36 mm head diameters prosthesis operated from the antero-lateral approach. In our study, participants treated with use of large femoral heads had significantly better gait patterns. Despite the quite small number of participants included in this study, a conclusion could be drawn that large heads used in THA seem to have an impact on gait restoration, making it more similar to the healthy participants’ one.

Results of this study might opt for performing THA with use of 36 mm heads. Even though there are several studies and systematic reviews concentrating on outcome, wear and revision rate in use of different femoral head sizes, more studies should analyse gait patterns following total hip replacement in terms of different implants, implant positioning or surgical approach.

Our study seems to be the first ever matched-cohort trial to analyse differences in functional outcome between different head sizes in patients operated from the anterolateral approach. Because of that, it can give a broader view on restoring function of affected legs which may be important in making decisions about operation. Our observations should make surgeons operating from anterolateral approach and using small femoral head sizes reconsider their decisions about implant parameters and in this case try to use larger femoral heads. What is more it can also be helpful information for physiotherapists, since patients operated from anterolateral approach with larger femoral heads used may achieve better functional results so there might be an indication to introduce specific exercises and rehabilitation protocols.

Furthermore, use of larger femoral heads could result in more physiological function of the hip and the whole limb, faster postoperative recovery, and increase patient’s satisfaction with the treatment.

In the future, next high-quality studies with randomization and multi-center studies should be performed to assess gait differences between uses of different femoral head diameters in total hip replacement. What is more, rehabilitation protocols following THR should be more emphasized in the literature to elaborate standardized, high-quality physiotherapy to restore gait pattern from the pre-disease period and improve functional outcome after THR. It might quicken returning to health, normal life functioning and potentially lower the number of revision surgeries.

## Data Availability

The datasets used and/or analysed during the current study will be available from the corresponding author on reasonable request.
